# Fulminating Postcaesarean Necrotising Fasciitis: A Rare and Lethal Condition Successfully Managed in a Resource-Disadvantaged Setting in Sub-Saharan Africa

**DOI:** 10.1155/2017/9763470

**Published:** 2017-05-16

**Authors:** Carlson-Babila Sama, Conrad S. Tankou, Fru F. Angwafo III

**Affiliations:** ^1^Bambalang Medicalized Health Centre, Bambalang, Cameroon; ^2^Galactic Corps Research Group (GCRG), Buea, Cameroon; ^3^Gynaeco-Obstetric and Paediatric Hospital and Department of Surgery, University Teaching Hospital, Yaoundé, Cameroon

## Abstract

Necrotising fasciitis is a rare but potentially lethal condition in obstetrics which usually presents with fulminant tissue destruction and a resultant high mortality. We report a 19-year-old Sub-Saharan female diagnosed with a rapidly erosive necrotising fasciitis on day 5 after caesarean section in a resource-limited setting. Timely diagnosis, aggressive antibiotic therapy, and prompt surgical intervention via an extensive abdominal wall debridement were pivotal to her survival.

## 1. Introduction

Necrotising fasciitis (NF) is a severe infection involving the skin, subcutaneous tissue, and superficial fascia, which often has a fulminant course and a potentially lethal outcome [1–5]. It is a rare infection with an estimated incidence of 0.4 per 100,000 in the general population and a mortality rate of up to 34% [5, 6]. Majority of cases result from trivial trauma; however, surgery is an independent risk factor [4, 5, 7]. The microbiology of wound infections resulting in necrotising fasciitis (types I–III) is primarily polymicrobial, cutting across a wide range of highly virulent pathogens as detailed in a recent review by Hakkarainen et al. [5]. However, Methicillin-resistant* Staphylococcus aureus* (MRSA) has been implicated in up to a third of cases [8].

Necrotising fasciitis had been earlier described by Hippocrates in the 5th century BC [9], but its association with caesarean section was first reported by Gretz et al. in 1962 [10]. Since then, Goepfert et al. [11] in an 8-year retrospective analysis of 5048 caesarean deliveries reported 9 cases, giving an incidence rate of 1.8 per 1000 caesarean deliveries while Schorge and colleagues [12] recorded five cases over a 15-year period.

Though a multidisciplinary team is best suited to handle such cases [3, 5], there is still an invaluable need to rely on general practitioners to perform a number of procedures in our setting where specialists are rare [13]. We herein report to the best of our knowledge the first case of widespread postcaesarean necrotising fasciitis successfully managed in a resource-limited primary health care centre in rural Cameroon with acceptable cosmetic results.

## 2. Case Presentation

We report a 19-year-old lady with a previous vaginal delivery who underwent a lower segment caesarean section (CS) for acute foetal distress at 39-week gestation. She delivered a healthy male baby and the surgery lasted 43 minutes. There were no intraoperative complications. She has no known medical condition and had an uncomplicated antenatal course.

**Figure 1:**
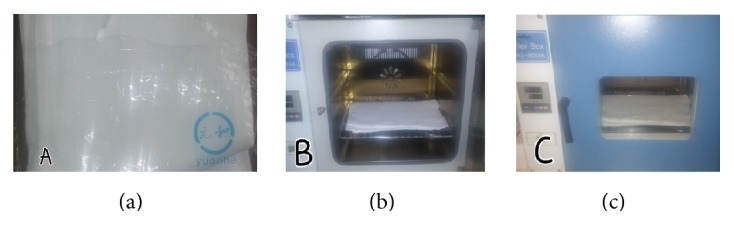
(a) Shows locally available baby napkin (diaper) in its original package; (b) shows some napkins placed in an autoclave; and (c) Depicts napkins undergoing the sterilisation process.

On day 4 after CS, she complained of lower abdominal pains. On examination, she was afebrile (temperature 37.1°C); other vital signs were within normal ranges and the incision site was clean with no evidence of surgical site infection. She was managed with analgesics (tramadol) and ampicillin was continued as part of her routine postoperative care. By day 5 postop, she developed an acute abdomen. The incision site had become very tender and soft tissue crepitation was noted on either side of the incision. Due to limited resources, only a white cell count, HIV status, random blood glucose, and a haemoglobin level could be done whose values were 39.800 cells/mm^3^, HIV negative, 116 mg/dL, and 8.3 g/dL, respectively. A decision to do an exploratory laparotomy was taken. Upon reopening the lower segment incision (Pfannenstiel), a pungent and offensive greenish seropurulent discharge oozed from the site. Further exploration revealed abundant foul smelling ascites and an extensive spread of greyish necrotic tissues involving the skin, subcutaneous tissue, recti muscles, fascia, and anterior serosa of myometrium. The hysterotomy site was nondraining and hyperaemic. An intraoperative diagnosis of necrotising fasciitis was made and all necrotic tissues were excised; the peritoneal cavity was thoroughly washed out and mopped using an adapted abdominal mop due to unavailability of a suction machine. This “abdominal mop” consisted of a commercially available baby napkin (diaper) made of cloth which was sterilised in an autoclave before it was used (Figure 1). Two locally adapted drains (drip lines attached to a urine bag) were also inserted. The resulting anterior abdominal wound was left open to heal by secondary intention and she was transfused a pint of whole blood in the immediate postoperative period. No samples were sent for analyses due to financial constraints. Empiric broad spectrum intravenous antibiotics (metronidazole, ceftriaxone, and gentamycin) were added and appropriate fluid support was also provided. Daily wound care was done during which necrotic tissues in the anterior abdominal wall were debrided to a bleeding edge.

**Figure 2:**
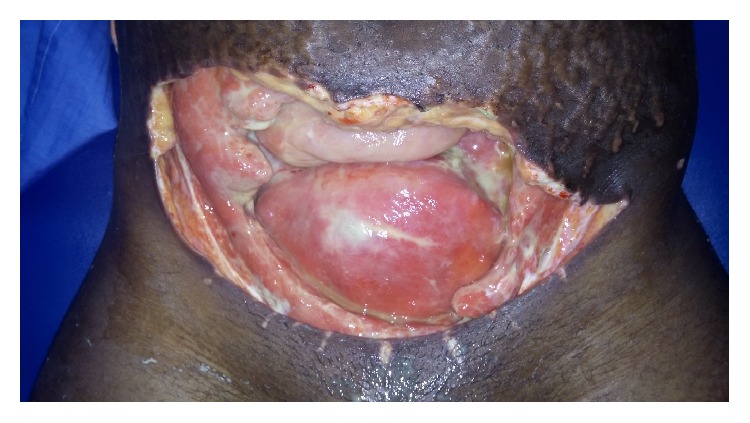
Large abdominal defect exposing bowel and uterus as seen on day 10 after extensive debridement.

**Figure 3:**
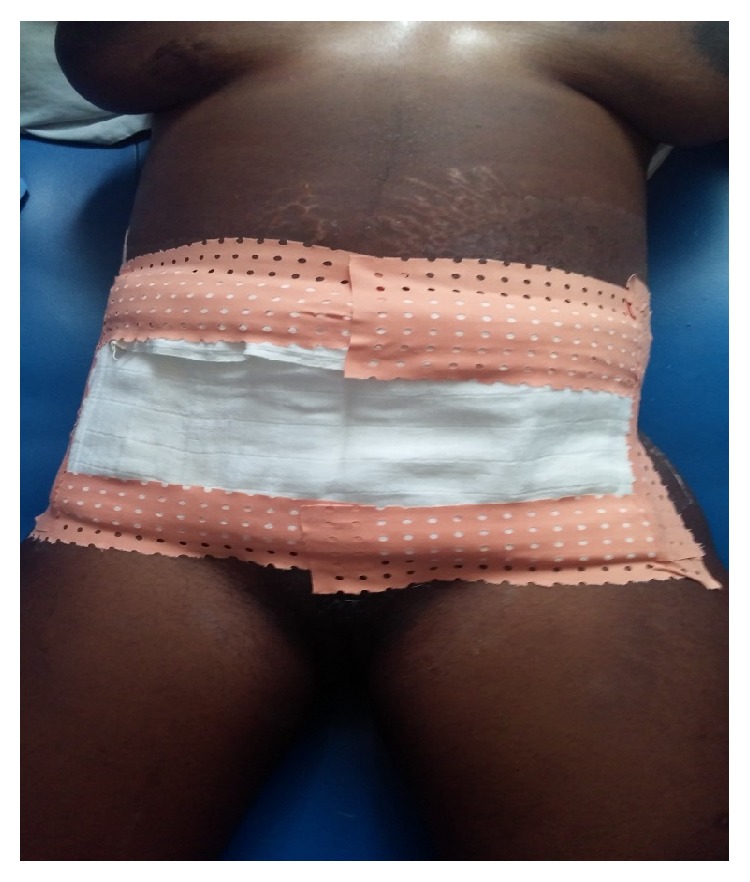
Sterilised baby napkin (diaper) used in covering abdominal defect after each dressing.

However, due to a deteriorating clinical state (septic shock: managed by continuation of empiric antibiotics and IV crystalloids: normal saline and Hartmann's solutions), abundant drainage, and continual progression of necrosis, the wound was extensively debrided approximately 2 cm beyond the bleeding edges to ensure adequate resection on day 6 after laparotomy. The large defect (Figure 2) with neither recti muscles nor fascia was covered with the improvised abdominal mop after each daily wound dressing (Figure 3). During this time, her baby was doing quite well and for fear of transmitting the infection to the infant, the child was often times separated from the mother and was bottle-fed with expressed breast milk.

**Figure 4:**
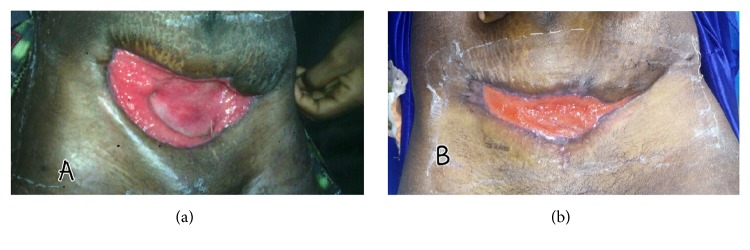
(a) and (b) Depicting wound healing process at weeks 15 and 22, respectively.

**Figure 5:**
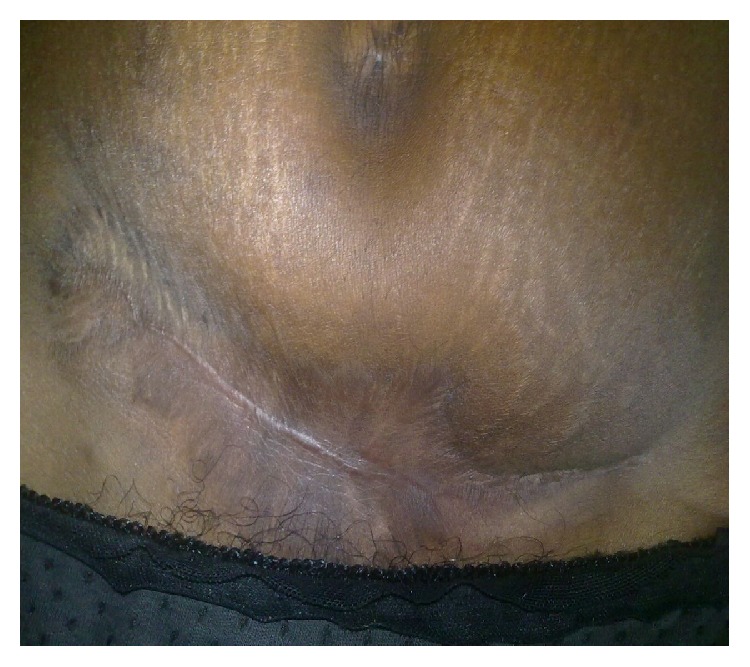
Completely healed anterior abdominal wall with appreciable cosmetics.

Once adequate margins on her anterior abdominal wall were obtained, evolution was remarkable as routine daily wound care did not show further necrosis, and there were decreased drainage and a marked improvement in her overall clinical status. In addition to her regular daily meals, nutritional support with* Glycine max *(in undocumented quantities) was provided (oral consumption). Two weeks after extensive debridement, a granulating and healthy looking wound was seen and regular breastfeeding had resumed. By the 15th week, the abdominal defect had closed and intraabdominal organs were nonvisible (Figure 4). She was discharged to be followed up on an outpatient basis; however, due to financial constraints regarding her bills, she remained in the health facility. Without any reconstructive surgery, complete closure of wound was achieved by week 25 after extensive debridement with appreciable cosmetics (Figure 5).

## 3. Discussion

Necrotising fasciitis (NF), also referred to as the “flesh-eating disease,” is a life-threatening infection which is usually polymicrobial in nature and requires timely surgical intervention and aggressive antibiotic therapy. It is a rapidly spreading infection and can involve all the layers of the soft tissue compartments as well as deeper tissues in the pelvis [3, 14–16]. Risk factors include diabetes, hypertension, anaemia, malnutrition, surgery, obesity, immunosuppression, peripheral vascular disease, alcoholism, smoking, and renal disease [3–5, 7, 14–17]. The condition is quite rare in obstetric literature with only about half a dozen cases reported in the last decade and it is often associated with high morbidity and mortality even with optimal treatment in specialised units, especially with infections extending to the pelvis [3, 4, 15–17] as in the current case report.

Clinically, most patients present with a dusky looking wound margin, bullae, crepitus, and signs of inflammation including swelling, induration (which may be difficult to recognize in dark skin patients), and severe pain disproportionate to local findings at the affected site [3–5, 7, 15–17]. Typically, the infection spreads along fascia planes due to its less robust blood supply and the overlying tissue can appear unaffected as noted in this case report. It is this feature that renders NF particularly difficult to diagnose without surgical intervention [5, 7, 16]. Thus, a high index of suspicion is warranted for early recognition as it characteristically spreads rapidly and can lead to systemic toxicity with a resultant multiorgan dysfunction and consequent death [3–5, 7, 15–17]. Worth reiterating is the fact that though paraclinical examinations including imaging modalities may be helpful [7]; the definitive diagnosis of NF is established surgically via direct visualization of fascia planes and muscle tissue [5, 16].

Whilst treatment with antibiotics plays an irreplaceable role in the management of NF, surgical intervention is the mainstay of management and should be done promptly as there is no room for “wait and watch policy” [3–5, 7, 16, 17]. Antibiotic therapy without debridement is associated with a mortality rate approaching 100% [5, 18]. As observed in this case report and similar published literature [3–5, 7, 15, 16], debridement may be serial and leaving the abdomen open is consistent with damage control laparotomy techniques which also has an added advantage of preventing abdominal compartment syndrome. Specialised materials like the Bogotá bag have been used to protect the abdominal viscera especially the bowels in such cases [16]; however, an improvised cloth (sterilised baby napkin) was effective in our resource-constraint setting. Once the infection has been cleared, a number of reconstructive surgical procedures can be done to ensure complete wound closure and enhance cosmetic appearance [3, 15].

This case underscores the importance of a high index of suspicion and prompt surgical intervention, even in the absence of hallmark features in managing necrotising fasciitis. Whilst not undermining the paramount importance of a highly specialised multidisciplinary team in managing such rare life-threatening conditions in sophisticated units, we have reported a successful management by primary health care physicians (general practitioners) with locally adapted materials in a resource-limited setting where referrals to tertiary centres seems a death sentence due to the associated unbearable financial burden. Though it is recommended that antibiotic treatment be adjusted according to microbiological findings, the role of empiric broad spectrum antibiotics in controlling the spread of the infection in such settings cannot be overemphasized. Nutrition with cost-effective and readily available food supplements like soya bean (*Glycine max: *38–45% protein content) is an effective adjunct to enhance healing. Our case management may not be standard, but it is subject to refinement and sets the platform for improvising in the management of various pathologies in the many health care-deprived resource-disadvantaged communities in Sub-Saharan Africa with little or no access to specialised care.

## References

[1] Headley A. J. (2003). Necrotizing soft tissue infections: a primary care review. *American Family Physician*.

[2] Wong C.-H., Chang H.-C., Pasupathy S., Khin L.-W., Tan J.-L., Low C.-O. (2003). Necrotizing fasciitis: clinical presentation, microbiology, and determinants of mortality. *The Journal of Bone & Joint Surgery—American Volume*.

[3] Rimawi B. H., Graybill W., Pierce J. Y. (2014). Necrotizing fasciitis and toxic shock syndrome from clostridium septicum following a term cesarean delivery. *Case Reports in Obstetrics and Gynecology*.

[4] Chhetry M., Banerjee B., Subedi S., Koirala A. (2016). Necrotizing fasciitis of anterior abdominal wall following cesarean section in a low-risk patient. *Journal of Surgical Case Reports*.

[5] Hakkarainen T. W., Kopari N. M., Pham T. N., Evans H. L. (2014). Necrotizing soft tissue infections: review and current concepts in treatment, systems of care, and outcomes. *Current Problems in Surgery*.

[6] Kaul R., McGeer A., Low D. E. (1997). Population-based surveillance for group A streptococcal necrotizing fasciitis: clinical features, prognostic indicators, and microbiologic analysis of seventy-seven cases. Ontario Group A Streptococcal Study. *American Journal of Medicine*.

[7] DeMuro J., Hanna A., Chalas E., Cunha B. (2012). Polymicrobial abdominal wall necrotizing fasciitis after cesarean section. *Journal of Surgical Case Reports*.

[8] Maya S. P., Beltrán D. D., Lemercier P., Leiva-Salinas C. (2014). Necrotizing fasciitis: an urgent diagnosis. *Skeletal Radiology*.

[9] Descamps V., Aitken J., Lee M. (1994). Hippocrates on necrotising fasciitis. *The Lancet*.

[10] Gretz H. F., Marchbanks V. H., Houle D. B. (1962). Progressive bacterial synergistic symbiotic gangrene. A complication of cesarean section. *American journal of obstetrics and gynecology*.

[11] Goepfert A. R., Guinn D. A., Andrews W. W., Hauth J. C. (1997). Necrotizing fasciitis after cesarean delivery. *Obstetrics and Gynecology*.

[12] Schorge J. O., Granter S. R., Lerner L. H., Feldman S. (1998). Postpartum and vulvar necrotizing fasciitis. Early clinical diagnosis and histopathologic correlation. *The Journal of Reproductive Medicine*.

[13] Mefire A., Atashili J., Mbuagbaw J. (2013). Pattern of surgical practice in a Regional Hospital in Cameroon and implications for training. *World Journal of Surgery*.

[14] Gallup D. G., Freedman M. A., Meguiar R. V., Freedman S. N., Nolan T. E. (2002). Necrotizing fasciitis in gynecologic and obstetric patients: a surgical emergency. *American Journal of Obstetrics and Gynecology*.

[15] Schumacher H., Tehrani H., Irwin M. S., Malata C. M. (2008). Abdominoplasty as an adjunct to the management of peri-Caesarian section necrotising fasciitis. *Journal of Plastic, Reconstructive and Aesthetic Surgery*.

[16] Uwizeyemariya C., Aboussou N., Dougnon S., Baidada A. (2015). Necrotizing fasciitis post cesarean section: case report and literature review. *International Journal of Recent Scientific Research*.

[17] Kulkarni T., Arora R., Looker N. (2007). Post-caesarean Meleney's gangrene revisited. *Journal of Obstetrics and Gynaecology*.

[18] Anaya D. A., Dellinger E. P. (2007). Necrotizing soft-tissue infection: diagnosis and management. *Clinical Infectious Diseases*.

